# Contact-Dependent Antibacterial Performance of Silver Nanoparticles Encapsulated in Collagen-Based Gels

**DOI:** 10.3390/jfb17030120

**Published:** 2026-03-02

**Authors:** Anastasia Ntolia, Nikolaos Matisioudis, Evgenia Dimitriou, Katerina Rouptsiou, Theofania Chatzigiannakou, Chris Manglaris, Michail Kalis, Eleftherios Triantafillou, Grammato Evangelopoulou, Maria Liakopoulou-Kyriakides, Vassilios Zaspalis, Nikolaos Michailidis, Amalia Aggeli

**Affiliations:** 1 Department of Chemical Engineering, Faculty of Engineering, Aristotle University of Thessaloniki, 57001 Thessaloniki, Greece; antoliac@cheng.auth.gr (A.N.); nmati@seas.upenn.edu (N.M.); s243214@dtu.dk (T.C.); cmanglaa@cheng.auth.gr (C.M.); michakalis@cheng.auth.gr (M.K.); mliako@cheng.auth.gr (M.L.-K.); zaspalis@auth.gr (V.Z.); 2 Department of Mechanical Engineering, Faculty of Engineering, Aristotle University of Thessaloniki, 57001 Thessaloniki, Greece; evgeniaod@auth.gr (E.D.); nmichail@meng.auth.gr (N.M.); 3 Hellenic Army, 41334 Larissa, Greece

**Keywords:** silver nanoparticles, antibacterial properties, collagen gels, biomedical applications

## Abstract

The design of new medical devices in biomedical engineering often necessitates the control of microbial load at the point of application, making antibacterial action valuable for numerous applications in the biomedical field. Nanotechnology products, such as silver nanoparticles (*AgNPs*), represent highly promising yet underexplored bioactive and antimicrobial agents that have attracted researchers’ interest for integration into medical devices. This study focuses on stable suspensions of silver nanoparticles, characterized by using a range of complementary physicochemical techniques as well as bacterial cell cultures, while also demonstrating controlled entrapment of the nanoparticles in collagen-based gels. The findings reveal that highly stable suspensions of negatively charged *AgNPs* (~6 nm in size) consistently exhibit broad-spectrum antimicrobial activity against both Gram-negative and Gram-positive bacteria, with minimum inhibitory concentration values of 10–20 ppm, whilst, importantly, close contact between the nanoparticles and bacterial cells turns out to be essential for their antibacterial action. Controlled entrapment of the nanoparticles in collagen-based gels enables regulation of nanoparticle release and their antimicrobial efficacy. This work highlights the promising prospects of silver nanoparticles in designing novel biomedical engineering products, while underscoring the need for a more comprehensive understanding of their biological activity to ensure optimal utilization.

## 1. Introduction

In biomedical engineering, an important design principle for the fabrication of medical devices is the ability to control the microbial load at the point of application, making antimicrobial performance valuable for numerous applications in the biomedical field. One of the most clinically established applications of antimicrobial medical devices lies in skin graft and wound management, for example advanced antimicrobial dressings offer the potential to reduce biofilm formation, which is a major barrier to healing, offering promise for next-generation wound care solutions [1,2]. Stents and other tubular devices used in cardiovascular or urinary applications are another area where antimicrobial properties have clear benefits. Stents are especially vulnerable to biofilm formation, which can lead to chronic infections and device failure. Research on novel stents modified with antimicrobial agents highlights how surface engineering can prevent microbial colonization and reduce the risk of infection without the need for systemic antibiotics [3]. For implantable devices, antimicrobial surface coatings are a major focus of current biomedical research. Biofilm-associated infections are a leading cause of implant failure, particularly for orthopedic, dental, and cardiovascular implants. By applying antimicrobial coatings, the surface of an implant can actively suppress bacterial adhesion and growth and protect itself. These coatings have been shown to extend device lifespan, lower infection rates, and reduce the need for revision surgeries [4,5,6]. Taken together, these diverse approaches to incorporating antimicrobial properties into medical devices underscore their broad applicability across device types and clinical contexts. By preventing infection at the interface between device and host, antimicrobial medical devices improve patient outcomes, reduce healthcare-associated infection rates, and support the long-term success of biomedical interventions.

Nanotechnology products, such as silver nanoparticles (*AgNPs*), represent highly promising yet underexplored bioactive antimicrobial agents that have attracted researchers’ interest for integration into medical devices. Furthermore, due to the global rise in multidrug resistance among microorganisms, there is an urgent need for the development of new and effective antimicrobial agents [7]. To address microbial resistance, new strategies have been designed that leverage nanotechnology [8,9]. Healthcare and medicine are the sectors with the most extensive research and development of technologies utilizing the antimicrobial activity of nanoparticles [10]. Nanoparticles present unique chemical, magnetic, mechanical, and biological properties that can modulate biocompatibility and cellular uptake [11]. Their small size allows them to circulate more freely within the human body. They can form complexes to serve as carriers for drugs or antibiotics [12], improving their release and selectivity, while other nanosystems demonstrate enhanced antimicrobial performance [13,14,15]. Metallic nanoparticles, such as silver, gold, and platinum, demonstrate promising antibacterial and antiviral effects [16,17,18]. The current state-of-the-art explores the beneficial combination of antimicrobial nanoparticles with medical devices [19,20,21,22,23,24], in an effort to reduce or even eliminate microbial contamination and colonization processes [25].

Silver, in all its forms, has historically been used as an antimicrobial agent, either on its own or in combination with other technologies [26]. This metal has been studied for its ability to inhibit bacterial growth, being incorporated as silver nitrate or silver sulfadiazine in creams and dressings for the treatment of burns and ulcers, in food packaging to prevent contamination and in household appliances such as refrigerators and washing machines [27,28,29,30,31,32]. Silver nanoparticles have also emerged as another form of antimicrobial silver; they are known to be the most extensively used nanoparticles in consumer products due to their widespread application as an antimicrobial agent [33,34].

Regarding the actual mechanistic aspects of the antibacterial activity of silver nanoparticles, there is a growing debate among scientists. Numerous studies have experimentally demonstrated that the anti-pathogenic activity of silver nanoparticles is superior to that exhibited by silver ions [35]. Silver nanoparticles display their intrinsically enhanced antimicrobial activity through various mechanisms [36], while simultaneously entering the biological system through different pathways. The routes of exposure, time, size, and state of aggregation, as well as the doses of silver nanoparticles, are associated with their bioavailability, biodistribution, and pathological symptoms [37].

Currently, the literature primarily supports three mechanisms, observed either together or separately, through which silver nanoparticles exert their antibacterial action [36,38,39,40,41,42]. The first mechanism suggests that silver nanoparticles act at the cell membrane level, as they are capable of penetrating the outer membrane, interacting with sulfur-containing proteins in the bacterial cell wall, and accumulating in the inner membrane. There, the adhesion of the nanoparticles to the cell membrane causes destabilization and damage, increasing membrane permeability and inducing leakage of cellular contents, ultimately leading to cell death [43,44]. The second mechanism proposes that the nanoparticles disrupt and traverse the cell membrane, altering its structure and permeability upon entering the cell but without seriously damaging the cell; once inside, it is suggested that silver nanoparticles have an affinity to interact with sulfur or phosphorus groups found in crucial intracellular components such as DNA and proteins, altering their structure and functions. Additionally, they may modify the respiratory chain in the inner membrane by interacting with thiol groups of the enzymes, inducing the production of reactive oxygen species and free radicals, thereby damaging the intracellular machinery and activating the apoptosis pathway. A third mechanism, proposed to work in tandem with the other two, is the release of silver ions from the nanoparticles; these ions can interact with cellular components, altering metabolic pathways, membranes, and even genetic material [44,45,46,47,48,49]. The behavior of Ag species (Ag^0^Nps/Ag^+^ ions) in aqueous biological media in which their antibacterial activity has been tested, still remains unclear. Due to the widespread presence of O_2_ in most aqueous biological media, the oxidative dissolution of Ag^+^ ions from Ag nanoparticles cannot be ignored. It has been reported that Ag nanoparticles showed much higher bactericidal activity in aerobic conditions as compared to anaerobic conditions [50]. This suggests that antibacterial action is actually due to Ag^+^ ions, and Ag nanoparticles only act as a reservoir and continuously release Ag^+^ ions due to their oxidative dissolution [41,43,51,52,53], this can result in the development of bacterial resistance to *AgNPs* [54]. Also, the recent literature indicates that the rate of dissolution of Ag^+^ ions from the surface of *AgNPs* plays an important role in their antibacterial activity [51].

*AgNPs* have been reported to have an effect against planktonic, biofilm-organized microorganisms, Gram-negative and Gram-positive bacteria, although Gram-positive bacteria tend to be less sensitive to the action of AgNPs than Gram-negative ones [55,56]. This is attributed to the difference in the Gram-negative and Gram-positive bacteria cell membranes: Gram-negative bacteria have a thin cell membrane (8–12 nm) [57], while Gram-positive bacteria have a thicker cell membrane (20–80 nm) that can be an obstacle for *AgNPs*’ penetration [58,59].

There is a wide range of *AgNPs* synthesized with different methods and decorated with different organic surface stabilizers, such as citrates, polyvinylpyrrolidone and polyvinyl alcohol [23], ending up with a wide range of different properties that affect their bioactivity. The size [23], shape [25], concentration [60], surface charge [61], and colloidal state [62] are the most important physicochemical parameters that affect the antimicrobial effects exhibited by *AgNPs*. Among various morphologies of silver nanoparticles (AgNPs)—spherical, triangular, linear, and cubic—spherical *AgNPs* consistently exhibit superior antibacterial efficacy [63]. The stability of the nanoparticle dispersion is another important factor affecting the final antibacterial activity [63]; when synthesized *AgNPs* exhibit low stability, they tend to aggregate into larger particles. As studies have demonstrated, larger or agglomerated nanoparticles display reduced antibacterial efficacy. Surface charge of the nanoparticles can also influence their antimicrobial activity, depending on the charge present on bacterial cell membranes [33,64,65,66]. Furthermore, regarding zeta potential, silver nanoparticles (*AgNPs*) are considered colloidally stable when their surface charge exceeds +30 mV or drops below −30 mV. This charge threshold promotes electrostatic repulsion between particles, effectively preventing agglomeration [41]. Notably, both the size and surface characteristics of nanoparticles significantly influence the rate at which silver ions are released. Smaller nanoparticles provide a greater contact area and enhanced interaction with the surrounding medium, whereas surface charge and composition play a key role in maintaining their overall stability [67]. Thus, smaller nanoparticles exhibit higher dissolution rates across various media, releasing silver ions that substantially contribute to their antibacterial efficacy [44,52,68]. Consequently, silver nanoparticles (*AgNPs*) smaller than 10 nm display markedly enhanced antimicrobial potency, owing to their larger surface area for silver ion release and increased interactions with bacterial cell membranes [69].

Despite the extensive theories that are available, the actual mechanisms of interaction that make *AgNPs* so efficient are still not clear due to the different processes competing in *AgNPs* activity, but also due to usually unstable, inhomogeneous or not well characterized *AgNPs*, as well as the poor understanding of incorporated *AgNPs* in biomedically relevant milieus. A primary challenge is the need to develop antimicrobial medical devices that effectively prevent infection while maintaining long-term biocompatibility and supporting tissue regeneration, particularly at the device–host interface [70,71,72].

In this context, collagen represents a highly advantageous option, as it is the most abundant structural protein in the human body and a fundamental component of the extracellular matrix, inherently promoting cell adhesion, migration, angiogenesis, and wound healing [73,74]. However, collagen-based biomedical devices remain vulnerable to microbial colonization and biofilm formation, creating a strong demand for antimicrobial strategies that do not compromise collagen’s native biological function [75]. Another major open issue concerns the controlled and predictable incorporation of antimicrobial nanoparticles, such as silver nanoparticles (*AgNPs*), within collagen matrices, as nanoparticle size, surface chemistry, and aggregation state strongly influence antimicrobial efficacy, cytocompatibility, and silver ion release kinetics under physiological conditions [70,76,77]. Furthermore, the relative contribution of *AgNP*-specific effects versus released Ag^+^ ions remains unclear, limiting rational product design and reproducibility across studies [78,79]. In addition, studies on the self-assembly of amphiphilic β-sheet peptide tapes into nanoscale structures provide foundational insights into the design of biomimetic scaffolds [80]. Finally, the lack of standardized in vitro and in vivo models that accurately replicate collagen-rich tissue environments—such as those addressed in nanobiotechnology peptide scaffolds—biofilm formation, and host immune responses continues to hinder clinical translation and regulatory approval of antimicrobial collagen-based biomedical devices [81,82,83]. In order to contribute to a better understanding and more efficient development of silver nanoparticle containing collagen-based medical devices, this study focuses on the entrapment and controlled release—controlled antibacterial performance of stable and well characterized silver nanoparticles in collagen-based gels; the study employs a wide range of complementary physicochemical techniques such as UV-Vis, DLS, zeta potential, TEM, SAED and rheology, as well as bacterial cell cultures of Gram-positive (*Staphylococcus aureus* and *Listeria monocytogenes*) and Gram-negative bacteria (*Escherichia coli* and *Salmonella typhimurium*) as a function of concentration of silver nanoparticles and initial bacterial load.

Despite extensive reports on AgNP–collagen antimicrobial systems, the dominant mechanism responsible for antibacterial efficacy remains ambiguous. In particular, it is rarely distinguished whether bacterial inhibition arises from released Ag^+^ ions, mobile nanoparticles capable of diffusing through the matrix, or immobilized AgNPs bound to the collagen network. This lack of mechanistic discrimination limits the rational design of biomedical products and leads to inconsistent performance across otherwise similar systems. The present study directly addresses this gap by experimentally isolating these contributions using collagen architectures that selectively enable or suppress nanoparticle mobility.

## 2. Materials and Methods

### 2.1. Materials

Silver nanoparticles were synthesized as described in [84] and they were stored in the dark as suspensions in deionized water at 300 ppm or 1500 ppm. Briefly, silver nitrate (99.9% AgNO_3_; M_r_ = 169.873 g/mol; Duchefa Biochemie, Haarlem, The Netherlands) was used as the silver precursor for AgNP synthesis, following a conventional literature-based reduction route. A protein stabilizer with a molecular mass of 20–25 kg/mol (Sigma-Aldrich, St. Louis, MI, USA) was added to control particle stabilization. Briefly, AgNPs were prepared via wet-chemical reduction by introducing the reducing agent into a preheated, stirred aqueous AgNO_3_ solution containing the stabilizer, ensuring complete dissolution prior to reduction. Gelatin (Sigma Aldrich Co. LLC., St. Louis, MI, USA), phosphate-buffered saline PBS (Sigma Aldrich Co. LLC., St. Louis, MI, USA), TSYEA (Tryptic Soy Yeast Extract Agar, BIOLIFE Italiana S.r.l., Bergamo, Italy), Nutrient broth (Bioprepare), Nutrient Agar (Bioprepare, Athens, Greece), *Escherichia coli* (WDCM 00090, ATCC 11775), *Staphylococcus aureus* (WDCM 00032), *Salmonella typhimurium* (NCTC 12023) and *Listeria monocytogenes* (NCTC 11994) were employed for the antibacterial experiments. The selection of these four test bacteria as representative Gram− and Gram+ microorganisms, was based on established laboratory expertise in handling these species, their well-characterized physiology, and consistent growth behavior under controlled experimental conditions, ensuring experimental reliability and accurate assessment of antibacterial activity and nanoparticle–bacteria interactions.

### 2.2. UV-Vis Spectrophotometry

Optical density was measured using two different UV-Vis spectrophotometers, 120-02 and 1800 Swimadzu, Kyoto, Japan in a range of 200–800 nm, with water or nutrient broth as the reference. Comparative spectra were collected without and with ultrasonication (30 min ultrasonication in ultrasonic water bath SONOREX RK 100 H, Berlin, Germany, without heating).

### 2.3. ζ-Potential

The ζ-potential measurement was performed on the Cordouan WALLIS Zeta Potential Analyzer Instrument, Pessac, France. The values of deionized water, i.e., viscosity (0.889 cP) and dielectric constant (78.06), were used in the settings. The temperature of the measurements was 25 °C.

### 2.4. Dynamic Light Scattering (DLS)

A VASCO DLS device (Cordouan Technologies, Pessac, France) was used to measure the distribution of hydrodynamic diameters of AgNPs. NanoQ software (version V2.6.2.0) package facilitated the controlling of hardware and analysis of results.

### 2.5. Transmission Electron Microscopy (TEM)

The size and morphology of the silver nanoparticles (AgNPs) were characterized using transmission electron microscopy (TEM). Sample preparation involved depositing an unstained droplet of the nanoparticle suspension onto a carbon-coated copper grid, followed by overnight drying. TEM analysis was conducted on a JEOL JEM-2011 HRTEM instrument, Tokyo, Japan, operating at an accelerating voltage of 200 kV.

### 2.6. Selected Area Electron Diffraction (SAED)

Once a region of interest was identified in bright-field imaging mode, the selected area aperture was inserted into the back focal plane to define the diffraction area (typically 200–1000 nm in diameter). The microscope was then switched to diffraction mode by adjusting the intermediate lens to the diffraction position, and the camera length was calibrated and set (e.g., 100–500 cm) to achieve the required reciprocal-space scale for accurate d-spacing measurements. The electron beam was centered and parallelized using the condenser and objective lenses. SAED pattern was captured digitally using a high-resolution CCD camera. Diffraction rings (polycrystalline) or spot patterns (single crystal) were indexed by measuring ring diameters or inter-spot distances with image analysis software (ImageJ version 1.53t). The resulting d-spacings were compared against reference powder diffraction data (e.g., ICDD PDF cards for face-centered cubic Ag) to assign Miller indices (*hkl*) and confirm the crystalline phase.

### 2.7. Rheology

A rheometer (AR-G2, TA Instruments, New Castle, DE, USA) using a 25 mm diameter sand blasted St–steel parallel plate geometry was used to carry out the rheological characterization, working in oscillation mode. The oscillation process was performed at temperature range 5–40 °C, ramp rate 1 °C/min, 3% strain, at a frequency of 0.1 Hz and delay time equal to 10 s. A Plexiglas cover over the geometry, with wetted cotton at its periphery, was used in all tests in order to reduce water evaporation into its enclosed volume. Equilibration of each sample on the rheometer took place for up to 15 min before the start of each measurement.

### 2.8. Collagen-Based Gel Preparation

Collagen gels at a concentration of 25% (*w*/*v*) were prepared in phosphate-buffered saline (PBS) in the presence of increasing concentrations of silver nanoparticles (*AgNPs*), with or without 1% glutaraldehyde. For the preparation of glutaraldehyde-crosslinked collagen gels, two different procedures were employed. In the first approach, covalent crosslinking of collagen via glutaraldehyde was performed prior to the incorporation of *AgNPs*, which were subsequently introduced into the gel by immersion. Under these conditions, the *AgNPs* were not permanently entrapped within the gel matrix and were free to diffuse out of the collagen network. In the second approach, glutaraldehyde was added in the presence of both collagen and *AgNPs*, resulting in covalent crosslinking not only between collagen chains but also between the *AgNPs* and collagen, thereby permanently entrapping the nanoparticles within the collagen gel.

### 2.9. Bacterial Growth Studies

Overnight cultures of the microbial strains were grown in Brain Heart Infusion Broth (BHI; BIOLIFE). To obtain the desired microbial populations, serial decimal dilutions were prepared in Maximum Recovery Medium (BIOLIFE). Initial populations of *Escherichia coli*, *Staphylococcus aureus*, *Listeria monocytogenes*, and *Salmonella typhimurium* (10^3^ and 10^6^ CFU/mL) were inoculated with silver nanoparticles at targeted final concentrations and incubated at 37 °C. Viable bacterial counts were determined at 0, 6, 24, and 48 h post-inoculation using the incorporation method on Tryptic Soy Yeast Extract Agar (TSYEA; BIOLIFE). Specifically, aliquots of bacterial suspensions were plated and incubated at 37 °C for 24–48 h, after which colony-forming units were enumerated. The same protocol was applied to the original silver nanoparticle solution to verify its sterility. The experiments were repeated with *Escherichia coli* suspensions at initial densities of 10, 10^2^, 10^3^, and 10^4^ CFU/mL in Nutrient Broth (Bioprepare), followed by incubation at 37 °C. Optical density measurements (OD_600_) were taken at 0, 48, 72, and 144 h. For the agar well diffusion assay, *E. coli* was grown on nutrient agar plates at 37 °C, and inhibition zones were subsequently measured.

Data were analyzed using IBM SPSS Statistics (version 29). one-way and two-way analyses of variance (ANOVA) were performed to evaluate the effect of *AgNP* concentration (25, 50, 100 ppm) and exposure time (0, 24, 48, and 72 h) on CFU/mL counts, which were treated as the dependent variables and with concentration and time treated as independent variables. Post hoc multiple comparisons were conducted using Tukey’s honestly significant difference (HSD) test to control for Type I error across pairwise comparisons. Homogeneity of variances was verified using Levene’s test. Statistical significance was set at α = 0.05.

## 3. Results and Discussion

### 3.1. Spectroscopic Analysis

**Figure 1:**
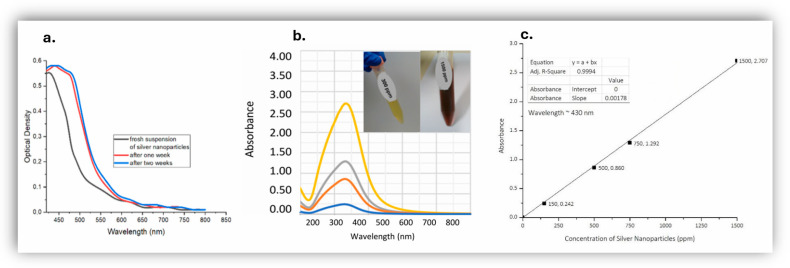
(**a**) UV-Vis spectra of a suspension of fresh silver nanoparticles 300 ppm with time, (**b**) UV-Vis spectra of suspensions of silver nanoparticles as a function of nanoparticle concentration (no prior ultrasonication), from bottom to top: 150 ppm (blue), 500 ppm (red), 750 ppm (gray) and 1500 ppm (orange), inset: photographs of vials of 300 ppm (left) and 1500 ppm (right) suspensions of nanoparticles in deionized water, (**c**) absorbance at maximum (*ca* 425 nm) as a function of silver nanoparticles’ concentration.

The aqueous suspensions of *AgNPs* seemed clear and had a characteristic yellow and brown color at 300 ppm and 1500 ppm respectively in deionized water with no visible precipitation or coagulation. They were initially studied by UV-Vis spectrophotometry, taking advantage of the distinctive surface plasmon resonance (SPR) phenomenon that takes place on the surface of silver nanoparticles [9]. Freshly prepared *AgNPs* were followed as a function of time with UV-Vis and the spectra were seen to stabilize after one week from synthesis of the nanoparticles (Figure 1a). Spectra that were collected from the same sample, even after a year from synthesis (stored in a sealed container in the dark at ambient temperature), were virtually identical (less than 10% difference) to the ones obtained after one week from synthesis. The UV-Vis spectra in deionized water as a function of silver nanoparticle concentration were characteristic of the presence of *AgNPs* due to a distinctive SPR peak centered at around 425 ± 5 nm (Figure 1b). It is known that the position of SPR peak shifts to higher wavelengths with the increasing size of nanoparticles, due to decreasing nanoparticle curvature which affects the transition energy and the wavelength at maximum absorbance of the observed SPR peaks [9]; a peak centered at 425 ± 5 nm, like the ones observed in this study (Figure 1b), corresponds to fairly small nanoparticles, with d < 50 nm [9]. The intensity at maximum absorbance was also found to be a linear function of increasing nanoparticle concentration up to at least 1500 ppm (Figure 1c). Comparative UV-Vis spectra of the samples were also collected after subjecting the samples to ultrasonication and near identical spectra were obtained before and after ultrasonication (Figure 1b,c and Supplementary Figure S1): the proportionality constant between UV-Vis intensity and nanoparticle concentration (in ppm) was found to be 0.00178 mL µgr^−1^ cm^−1^ without ultrasonication (Figure 1c) and 0.00175 mL µgr^−1^ cm^−1^ after ultrasonication (Supplementary Figure S1). Furthermore no peaks or increased intensity were ever observed in the range of 600–700 nm, which further corroborates the absence of nanoparticle agglomeration. Suspensions of silver nanoparticles in Nutrient Broth gave similar UV-Vis spectra as the ones in water (Supplementary Figure S1) and again no visual sign of aggregation.

### 3.2. ζ-Potential Studies

**Figure 2:**
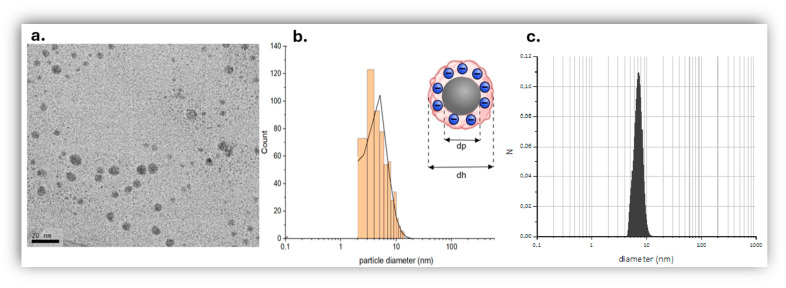
(**a**) Unstained TEM micrograph of silver nanoparticles, 300 ppm aqueous suspension diluted to 1:25 with distilled water prior to placement on the TEM grid, scale bar: 20 nm, (**b**) size distribution of nanoparticles in TEM by image analysis (log normal distribution), with a schematic representation of a silver nanoparticle as an inset, (**c**) DLS of a suspension 300 ppm *AgNPs* in deionized water.

To further characterize the nanoparticles, the zeta potential of *AgNPs* in deionized water was measured and determined on average to be −34.99 mV (Table 1). The electrophoretic mobility of the AgNPs was consistently negative (fitted mean −2.71 ± 0.67 μm·cm/V·s), in good agreement with the measured fitted zeta potential (−34.8 ± 8.6 mV), confirming a negatively charged and electrophoretically stable dispersion. The organic layer covering each nanoparticle seems to be negatively charged; this endows the suspension of silver nanoparticles with sufficient physical colloid stability due to interparticle electrostatic repulsions, thus creating stable suspensions of NP (Figure 2b) [66]. The net negative charge on the surface of *AgNPs* seems also enough to prevent them from aggregating even in Nutrient Broth given that the UV-Vis spectra of *AgNPs* in deionized water and in nutrient broth are similar (Figure 1 and Supplementary Figure S1).

### 3.3. TEM and DLS Analyses of Size Distribution

Unstained TEM micrographs revealed the presence of tiny, spherical nanoparticles (Figure 2a), whilst image analysis using ImageJ of several micrographs produced a size distribution (Figure 2b) centered at particle diameter dp of 5.9 ± 2.7 nm. Bulk DLS study of the hydrodynamic diameter dh of the nanoparticles was also carried out that showed a single peak centered at 7 nm (Figure 2c), in agreement with the TEM measurements (Figure 2b).

### 3.4. Selected Area Electron Diffraction (SAED) Analysis

**Figure 3:**
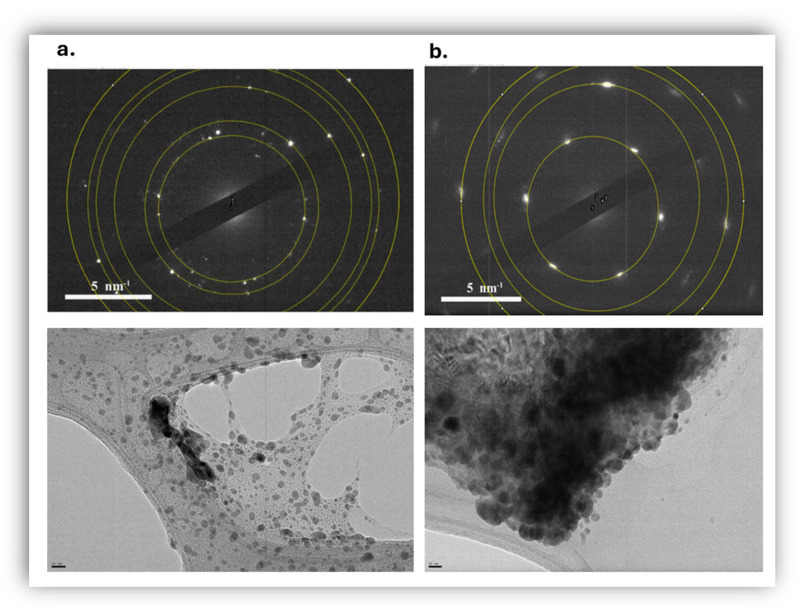
(**a**) SAED analysis of stable suspension of silver nanoparticles 300 ppm (TEM-left-scale bar 20 nm). (**b**) SAED analysis of precipitated silver nanoparticles (TEM-right-scale bar 20 nm).

Selected area electron diffraction was used in TEM, to analyze the crystal structure of silver nanoparticles (Figure 3); the selected area has more than one nanoparticle, so rings of spots rather than individual spots appear, due to the differently oriented crystal lattices. SAED analysis was carried out both in stable suspensions and in precipitated nanoparticles (in an attempt to increase the obtained signal).

It is evident that the nanoparticles are nanocrystalline, whilst Miller indices were determined in order to find out the crystal planes for each ring, by calculating the radius of the ring with the Fiji software (version 1.53t), which corresponds to the distance between the planes (d-spacing). Six ellipses were formed and the results of the values of the major and minor axes were used to calculate d-spacings which were compared with known data for FCC-Ag (ICDD PDF: 00-004-0783, International Center for Diffraction Data). The data correspond to silver crystallographic planes (111), (200), (220), (311), (222) and (400) and thus the crystal structure of *AgNPs* is face-centered cubic (FCC). Similar results were obtained in the stable and precipitated nanoparticles.

### 3.5. Bacterial Growth Kinetics in the Presence of AgNPs

Initially *AgNPs* suspensions were added in sterile growth media and upon incubation for up to 48 h, no growth of microorganisms was observed, which proved the inherent sterility of the *AgNPs* suspensions. Indeed aqueous suspensions of *AgNPs* with >10 ppm *AgNPs* have never been seen during this study, to ever be infected by bugs even after years of storage at room temperature.

**Figure 4:**
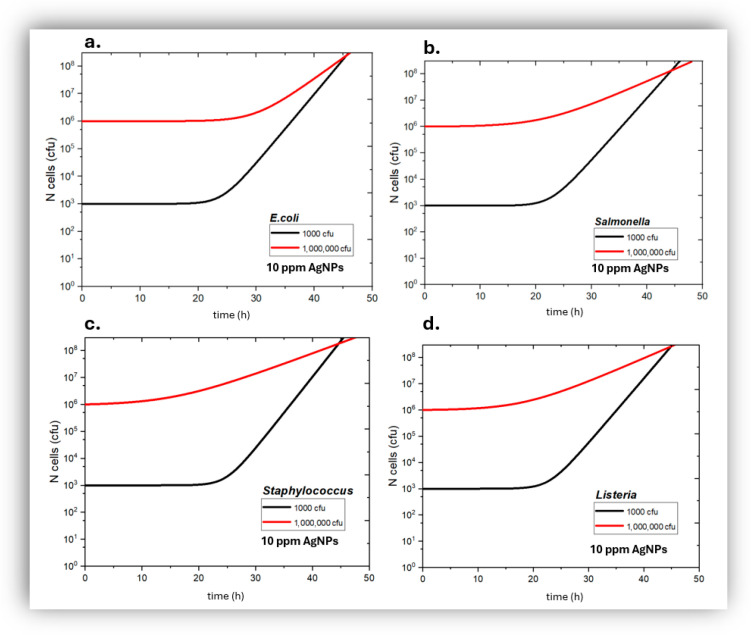
Growth kinetics of *Escherichia coli* (**a**), *Salmonella typhimurium* (**b**), *Staphylococcus aureus* (**c**), and Listeria *monocytogenes* (**d**), in the presence of 10 ppm of silver nanoparticles as a function of incubation time, for initially low (black) and high (red) bacterial load.

**Figure 5:**
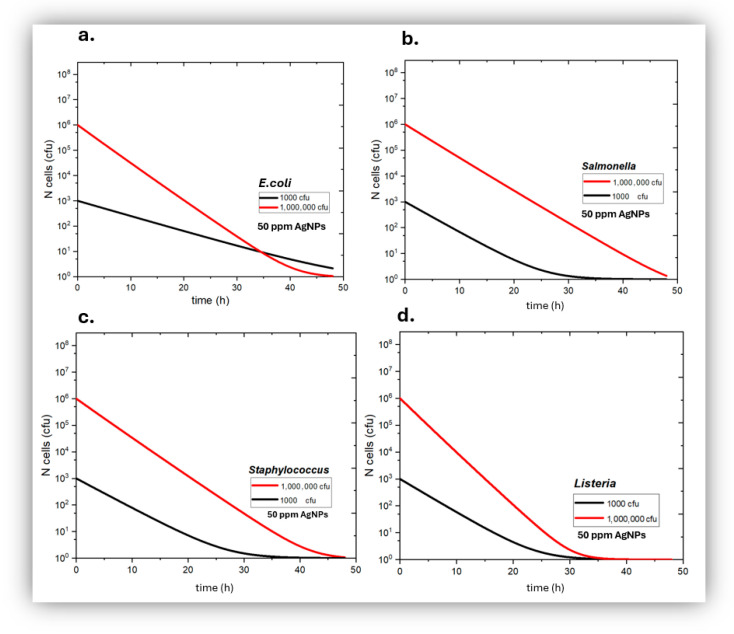
Growth kinetics of *Escherichia coli* (**a**), *Salmonella typhimurium* (**b**), *Staphylococcus aureus* (**c**) and *Listeria monocytogenes* (**d**), in the presence of 50 ppm of silver nanoparticles as a function of incubation time, for initially low (black) and high (red) bacterial load.

**Figure 6:**
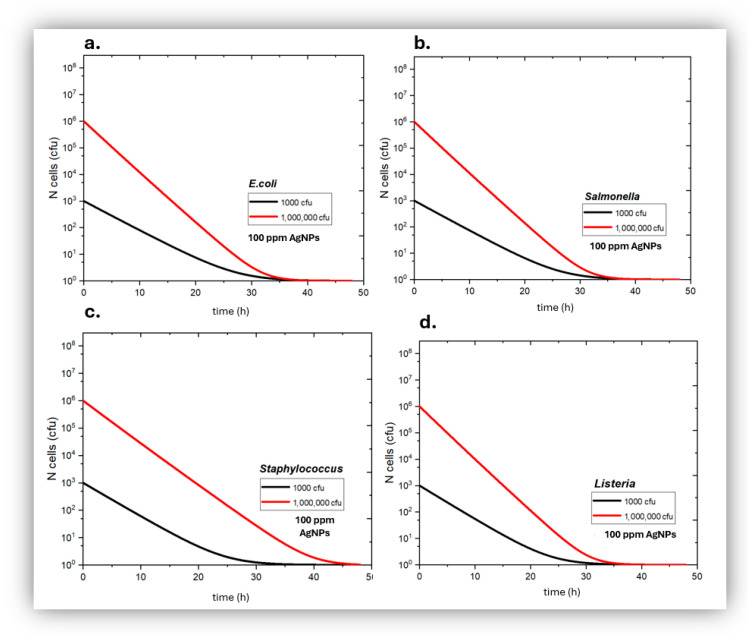
Growth kinetics of *Escherichia coli* (**a**), *Salmonella typhimurium* (**b**), *Staphylococcus aureus* (**c**), and *Listeria monocytogenes* (**d**), in the presence of 100 ppm of silver nanoparticles as a function of incubation time, for initially low (black) and high (red) bacterial load.

Quantitative in vitro experiments of bacterial growth kinetics were carried out with *Escherichia coli*, *Staphylococcus aureus*, *Salmonella typhimurium* and *Listeria monocytogenes* based on colonies’ counting on Petri dishes. The following Figure 4, Figure 5 and Figure 6 show the bactericidal action of silver nanoparticles as a function of *AgNP* concentration and incubation time for both low and high initial bacterial load. At 10 ppm (Figure 4) the *AgNPs* appear to have a bacteriostatic action for the first 24 h where the rate of bacterial death could be equal to the rate of bacterial growth, whilst afterwards both Gram− and Gram+ bacteria start showing a net growth again. This unexpected behavior suggests a possible reduction in antibacterial efficacy over time. A probable explanation is related to the gradual inactivation of *AgNPs* during prolonged incubation, which may arise from nanoparticle aggregation and/or interactions with constituents of the culture medium, all of which could significantly decrease the availability of bioactive silver species, leading to a loss of bactericidal activity and the observed bacterial regrowth. Bacterial adaptation could be another possible explanation for this phenomenon; however, the rather short incubation time of 24 h is unlikely to have allowed significant bacterial adaptation for all for bacterial species studied here at both low and high initial bacterial load.

At 50 ppm (Figure 5), the AgNPs showed a significant bactericidal action against all four bacterial types studied and for all conditions tried; bacterial population kept decreasing with time in contact with 50 ppm *AgNPs*; all bacterial samples of both Gram+ and Gram− bacteria, even those that had a high initial bacterial load, reached effective sterility after 48 h of exposure to 50 ppm *AgNPs*.

In the presence of 100 pm *AgNPs* (Figure 6), all obtained bacterial kinetics showed even stronger antibacterial action of *AgNPs*, compared to 50 ppm *AgNPs*; all samples tried reached effective sterility state after being exposed to 100 ppm *AgNPs* for <30 h.

**Figure 7:**
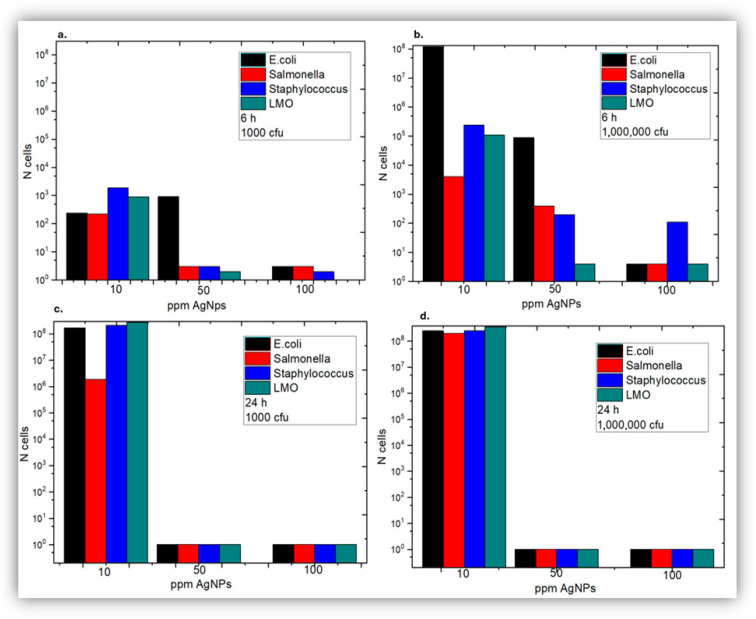
Comparative results of bacterial growth after 6 and 24 h of incubation in the presence of 10, 50 and 100 ppm silver nanoparticles: (**a**) Initial bacterial load of 10^3^ cfu, after 6 h. (**b**) Initial bacterial load of 10^6^ cfu, after 6 h. (**c**) Initial bacterial load of 10^3^ cfu, after 24 h. (**d**) Initial bacterial load of 10^6^ cfu, after 24 h.

The results were summarized in Figure 7, where it becomes clear that 10 ppm *AgNPs* do not create robust bactericidal conditions; on the other hand, 50 ppm and 100 ppm *AgNPs* are highly bactericidal. However 50 ppm *AgNPs* require at least 24 h exposure in order to control effectively bacterial load, whilst 100 ppm are highly effective even after 6 h of exposure due to faster bactericidal kinetics compared to 50 ppm *AgNPs*. Therefore the minimum inhibitory concentration MIC was placed at 10 ppm < MIC < 50 ppm. No significant changes were observed in Gram− versus Gram+ bacteria.

**Figure 8:**
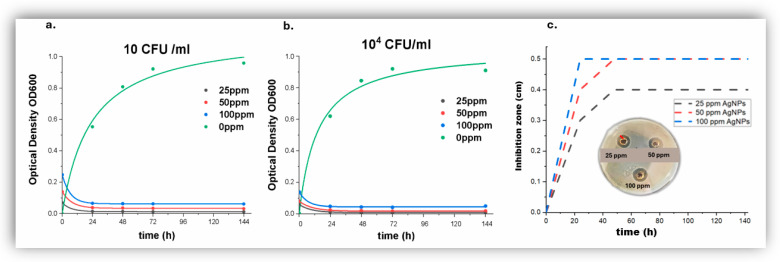
(**a**,**b**). Growth kinetics of *Escherichia coli* in liquid substrates incubated in the presence of 0, 25, 50 and 100 ppm *AgNPs*, as a function of initial bacterial load of 10 and 10,000 cfu mL^−1^, followed by OD at 600 nm. (**c**) Zone of inhibition over time for *AgNPs* suspensions of 25 (black), 50 (red) and 100 ppm (blue). Inset: zones of inhibition observed around wells containing *AgNPs* suspensions of 25, 50 and 100 ppm; the zone of inhibition that was measured corresponds to the distance between the edge of the well and the area of zero bacterial growth, as shown with the red line.

Repeat in vitro experiments were carried out with model *Escherichia coli* cultures in liquid substrate, where the effect of 25 ppm *AgNPs* was also studied in order to pinpoint the MIC value in more detail (Figure 8a,b and Supplementary Figure S2). Bacterial growth kinetic studies revealed that 25 ppm AgNPs are highly bactericidal for all starting bacterial loads of 10, 100, 1000 and 10,000 cfu mL^−1^.

A one-way ANOVA revealed a significant effect of AgNP concentration on bacterial growth, post hoc comparisons using the Tukey HSD test showed that the control group (0 ppm, M = 0.6735) differed significantly from all AgNPs groups (25 ppm: M = 0.0142, 50 ppm: M = 0.0318, 100 ppm: M = 0.0610, *p* < 0.05), while no significant differences were observed among the 25, 50, and 100 ppm groups (*p* = 0.690). Two-way analysis of variance (ANOVA) revealed a statistically significant effect of exposure time on optical density (OD_600_) (F = 31.058, *p* < 0.001, partial η^2^ = 0.705), as well as a significant effect of AAgNP concentration (F = 29.998, *p* < 0.001, partial η^2^ = 0.606). The interaction between the examined factors indicated that both time and silver nanoparticle concentration contribute substantially to the observed reduction in optical density. Post hoc analysis using Tukey’s HSD test demonstrated that OD_600_ values at 24 h, 48 h, and 72 h were significantly lower compared to 0 h (*p* < 0.001), whereas no statistically significant differences were detected among the 24 h, 48 h, and 72 h time points. This pattern suggests a sustained antimicrobial effect following the initial exposure period. The boxplot analysis further supports these findings, showing comparable dispersion and median values of optical density at 24 h, 48 h, and 72 h. The consistency of the distributions across these time points indicates a stable antimicrobial performance of AgNPs over time rather than a transient effect. As shown in Figure S8 (Supplementary Material), optical density decreases with increasing AgAgNP concentrationcross all exposure times, whereas Figure S9 (Supplementary Material), highlights the time-dependent reduction and subsequent stabilization of OD_600_ values. Moreover, a progressive decrease in optical density was observed with increasing AgNP concentration, with the highest concentration (100 ppm) exhibiting the strongest inhibitory effect. These results collectively demonstrate a time-dependent and concentration-dependent antimicrobial activity of silver nanoparticles, characterized by an early onset followed by sustained efficacy.

Furthermore complementary inhibition zone studies in bacterial cultures on solid substrates (Figure 8c and Supplementary Figure S2) showed that 25 ppm *AgNPs* produced significant zones of inhibition of bacterial growth and therefore it consolidated the strong antibacterial action of 25 ppm. Therefore the MIC value was found to be 10 ppm < MIC < 25 ppm.

**Figure 9:**
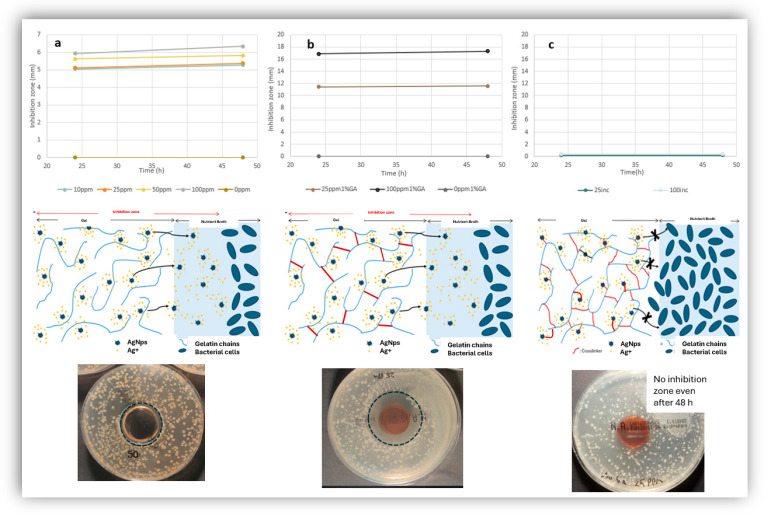
Antibacterial activity of *AgNPs* encapsulated in collagen gels. (**a**) Uncrosslinked collagen gels were produced in the presence of *AgNPs* and their antibacterial activity was studied as a function of *AgNP* concentration by measuring the zone of bacterial inhibition. (**b**) A second type of collagen gel was produced by chemical crosslinking of collagen chains with glutaraldehyde; they were then immersed in a *AgNP* suspension and incubated; they showed strong zones of bacterial inhibition that increased with *AgNP* concentration. (**c**) A third type of collagen gel was produced in which *AgNPs* were added during glutaraldehyde cross linking; therefore, the *AgNPs* were covalently and permanently bound inside the stable crosslinked collagen gel; these gels did not show any antibacterial activity (no zone of inhibition) even at the higher *AgNP* concentrations. These results demonstrate that antibacterial activity is strictly associated with nanoparticle mobility and direct bacterial contact.

**Figure 10:**
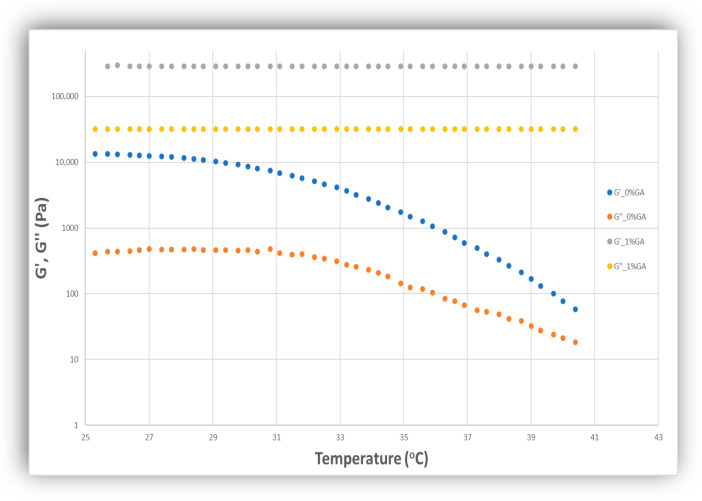
Rheological properties G′ and G″ as a function of temperature for uncrosslinked (G′ blue, G″ red) and crosslinked (G′ gray, G″ orange) collagen-based gels.

The silver nanoparticles were then encapsulated in collagen-based gels, relevant in the context of biomedical applications. Uncrosslinked collagen gels were produced in the presence of *AgNPs* and their antibacterial activity was studied as a function of *AgNP* concentration by measuring the zone of bacterial inhibition (Figure 9a). The gels gradually melted during incubation time, but they still exhibited mild zones of inhibition for >20 ppm *AgNPs* which increased with increasing *AAgNP* concentration Preliminary rheological analysis of these gels showed a G′ of *ca* 13,000 Pa and a G″ of *ca* 500 Pa for temperature lower than 30 °C and an abrupt decrease in the rheological properties above 30 °C (Figure 10) which explains the observed melting of the gels during incubation in bacterial cultures. Another set of collagen gels were produced by chemical crosslinking of collagen chains with glutaraldehyde. These were characterized by superior mechanical properties: a G′ of *ca* 290,000 Pa and a G″ of *ca* 32,000 Pa, more than an order of magnitude higher compared to uncross linked gels, at all temperatures studied (Figure 10). They were then immersed in a *AgNP* suspension and incubated; these gels were stiff and stable and did not melt during incubation, as expected based on their superior rheological properties which are largely insensitive to temperature in the range studied here; they also showed strong zones of bacterial inhibition that increased with *AgNP* concentration (Figure 9b). To directly discriminate between diffusion-mediated and immobilized *AgNP* antibacterial mechanisms, three collagen architectures were systematically compared. The antibacterial activity observed in Figure 9a,b could be due to either diffusion of released Ag^+^ ions or due to diffusion of *AgNPs*. In order to tell the difference between the two possibilities, a third type of collagen gel was produced in which *AgNPs* were added during glutaraldehyde cross linking; therefore the *AgNPs* were covalently and permanently bound inside the stable crosslinked collagen gel; interestingly these gels did not show any antibacterial activity (no zone of inhibition) even at the higher *AgNP* concentrations (Figure 9c). This is a strong indication that in order to exhibit their antibacterial action, the *AgNPs* need to diffuse until they reach the vicinity of the bacterial cells; *AgNPs* that cannot diffuse in order to reach near the bacterial cells do not have any measurable antibacterial activity in their surrounding environment. Control gels were also studied that had Ag+ encapsulated inside, rather than silver nanoparticles; under identical incubation conditions, no antimicrobial effect was observed up to 100 ppm AgNO_3_ which further strengthened the conclusion that for the silver nanoparticle concentration range studied here, the main antimicrobial effect is based on diffusion of nanoparticles rather than of silver ions.

### 3.6. Design Implications for Collagen-Based Antimicrobial Biomaterials

The present findings provide important design implications for the development of collagen-based antimicrobial biomaterials incorporating silver nanoparticles. While *AgNPs* are widely reported as effective antibacterial agents, their incorporation into collagen matrices is often treated as intrinsically sufficient to confer antimicrobial functionality. Our results demonstrate that this assumption is overly simplistic and may lead to inconsistent or misleading outcomes.

By experimentally isolating different *AgNP*–collagen architectures, we show that antibacterial efficacy is not governed by the mere presence of silver nanoparticles, but rather by the ability of nanoparticles to interact directly with bacterial cells. Specifically, collagen systems that permit nanoparticle mobility and diffusion exhibit pronounced antibacterial activity, whereas covalently immobilized *AgNPs* embedded within the collagen network show no measurable antibacterial effect, despite identical nanoparticle size and concentration. This distinction provides direct evidence that immobilization fundamentally alters the biological functionality of *AgNPs*. These observations establish a critical architecture-dependent design principle: collagen-based biomaterials containing immobilized silver nanoparticles should not be assumed to be antimicrobial. Instead, antibacterial performance requires either nanoparticle transport within the matrix or sufficiently close contact between mobile nanoparticles and bacterial cells. This finding helps rationalize the wide variability in reported antibacterial outcomes for superficially similar *AgNP*–collagen systems in the literature, where differences in crosslinking density, nanoparticle binding, or matrix permeability are often underreported.

From a materials engineering perspective, this mechanistic insight enables more rational design strategies. Systems intended for strong antibacterial performance must be engineered to allow controlled nanoparticle mobility or release, whereas applications prioritizing long-term stability or safety may deliberately favor immobilized architectures, accepting the absence of antibacterial activity. Importantly, these considerations highlight that “silver-containing” and “antimicrobial” are not interchangeable descriptors for collagen-based biomaterials. Overall, the present study shifts the focus from compositional selection toward structural and transport-based design, emphasizing that the biological function of *AgNP*-containing collagen materials is dictated by matrix architecture and nanoparticle accessibility. This framework provides a mechanistic basis for designing collagen-based antimicrobial biomaterials with predictable and reproducible performance.

### 3.7. Biocompatibility and Safety Considerations

While silver nanoparticles (*AgNPs*) are increasingly explored for antibacterial biomaterials and other biomedical engineering applications, their potential cytotoxicity and biosafety profiles remain an essential consideration. *AgNPs* can interact with biological systems in a dose-, size-, surface chemistry-, and exposure-dependent manner, exhibiting antimicrobial efficacy and, at higher exposures, toxic effects on mammalian cells and tissues [85,86]. Several comprehensive reviews and experimental studies confirm that cytotoxic mechanisms of *AgNPs* involve oxidative stress generation, disruption of mitochondrial function, and interference with cellular processes including DNA replication and protein synthesis [85,87]. The magnitude of these effects strongly correlates with particle size, concentration, and surface properties; smaller particles and higher dosages generally increase the likelihood of adverse cellular responses [86]. Importantly, surface functionalization and formulation strategies can mitigate cytotoxicity while preserving antibacterial performance. For example, coating *AgNPs* with biocompatible ligands such as cysteine or glutathione has been shown to reduce cellular toxicity relative to ionic silver forms, highlighting the role of nanoparticle design in biosafety [88]. Similarly, AgNP composites embedded in polymeric or hydrogel matrices have reported enhanced biocompatibility in vitro and in vivo, depending on *AgNP* concentration and matrix interactions [89]. Several studies specifically demonstrate that collagen-stabilized *AgNP* systems can support mammalian cell viability while maintaining antibacterial function, indicating that appropriate material design can help balance efficacy and safety [90]. Moreover, recent work emphasizes that immobilizing *AgNPs* within a matrix tends to limit systemic nanoparticle release and reduce potential cytotoxicity, thus aligning antibacterial performance with safer usage profiles for biomedical contexts.

In the present study, the antibacterial effects were observed within a relatively low *AgNP* concentration range (*ca* 25 ppm), which is consistent with ranges reported to achieve antibacterial potency with minimal adverse effects in cell-based assessments. Furthermore, our observation that covalently immobilized *AgNPs* lack measurable antibacterial activity—despite exposure to bacteria—suggests that particle mobility and direct contact are necessary for efficacy. Taken together, these findings and the supporting literature indicate that while cytotoxicity concerns for *AgNPs* should not be overlooked, rational nanoparticle design and matrix selection can provide a path toward safe and effective application of *AgNP*-based collagen biomaterials in biomedical engineering. Comprehensive preclinical evaluation and adherence to emerging nanosafety guidelines will remain crucial in advancing such materials toward clinical use.

## 4. Conclusions

Antibacterial action is useful for many applications in the biomedical area because the creation of new medical devices in biomedical engineering frequently requires the control of microbial load at the place of application. Researchers are interested in integrating nanotechnology products, including silver nanoparticles (*AgNPs*), into medical devices because they are highly promising but understudied bioactive and antibacterial agents [91,92,93]. In addition to exhibiting controlled entrapment of the nanoparticles in collagen-based gels, this work focuses on stable suspensions of silver nanoparticles, which are described using a variety of complementary physicochemical techniques and bacterial cell cultures. Importantly, close contact between the nanoparticles and bacterial cells turns out to be crucial for their antibacterial action. The results show that highly stable suspensions of negatively charged *AgNPs* (~6 nm in size) consistently exhibit broad-spectrum antimicrobial activity against both Gram-negative and Gram-positive bacteria, with minimum inhibitory concentration values of 10–20 ppm. In line with modern techniques that incorporate *AgNPs* into collagen nanofibers and gelatin materials, which improve wound healing, lower cytotoxicity, and promote bone regeneration, controlled entrapment of the nanoparticles in collagen-based gels allows regulation of their release and antimicrobial efficacy [94,95,96,97,98].

The face-centered cubic (FCC) crystalline structure and compact size (~6 nm) of the *AgNPs* developed herein closely match characteristics of biocompatible nanoparticles investigated for biomedical applications, such as wound dressings and controlled drug delivery. Systematic reviews confirm that nanoparticles of this scale exhibit limited cytotoxicity at modest concentrations across various cell lines—particularly green-synthesized variants stabilized by biocompatible coatings—due to reduced reactive oxygen species and mitochondrial disruption. These attributes bolster the feasibility of deploying our AgNPs in collagen matrices for enhanced wound healing, subject to optimized dosing [99]. Although ~6–10 nm *AgNPs* may penetrate cells more readily, studies report only mild effects for FCC-structured particles under controlled conditions. For instance, green-synthesized equivalents showed dose-dependent low toxicity in HEK-293 and RAW 264.7 cultures, with negligible viability impacts at therapeutically relevant levels. Future efforts could emphasize surface functionalization, as biologically coated *AgNPs* outperform chemical ones in biocompatibility [100]. This work highlights the promising prospects of silver nanoparticles in designing novel biomedical engineering products, while underscoring the need for a more comprehensive understanding of their biological activity to ensure optimal utilization.

## Figures and Tables

**Table 1:** Raw and fit values of potential (ζ and μ) of a 300 ppm aq. suspension of silver nanoparticles (pH = 4.5).

	Raw		Fit			
Νο	μ (μm·cm/V·s)	ζ (mV)	μ (μm·cm/V·s)	σ	ζ (mV)	Σ
1	−2.41	−31.02	−2.61	0.74	−33.52	9.53
2	−3.16	−40.56	−2.89	0.71	−37.16	9.14
3	−2.41	−31.02	−2.53	0.73	−32.47	9.45
4	−2.6	−33.4	−2.56	0.68	−32.95	8.8
5	−3.16	−40.56	−3.17	0.5	−40.72	6.49
6	−2.6	−33.4	−2.5	0.65	−32.09	8.39
**Avg**	**−2.72**	**−34.99**	**−2.71**	**0.67**	**−34.82**	**8.63**

## Data Availability

The original contributions presented in this study are included in the article. Further inquiries can be directed to the corresponding author.

## Supplementary Materials

The following supporting information can be downloaded at: https://www.mdpi.com/article/10.3390/jfb17030120/s1, Figure S1: (a) UV-Vis of 25, 50 ppm of suspension of silver nanoparticles in water and (b) UV-Vis of 25, 50 and 100 ppm of silver nanoparticles in nutrient broth. Figure S2: (a) UV-Vis of 150–1500 ppm suspension of silver nanoparticles after sonication, (b) Absorbance at maximum (*ca* 425 nm) as a function of silver nanoparticles’ concentration. Figure S3: (a) Growth kinetics of *E. coli* in liquid substrates, followed by optical density at 600 nm incubated in the presence of 25 ppm AgNPs, as a function of initial bacterial load, (b) Optical density over time for diluted *E. coli* culture with initial concentration 1000 cfu/mL with AgNPs 25, 50 and 100 ppm, (c) zone of inhibition in relation to concentration of AgNPs. Figure S4: Two-way analysis of variance (ANOVA) assessing the effects of AgNps concentration and exposure time on optical density (OD_600_). Type III sums of squares, F-values, significance levels (*p*-values), and partial eta squared (η^2^) are reported. Figure S5: Post hoc multiple comparisons of optical density (OD_600_) between exposure times using Tukey’s honestly significant difference (HSD) test following two-way ANOVA. Figure S6: Ranking of exposure times based on homogeneous subsets of optical density (OD_600_) identified by Tukey’s HSD test. Mean values are grouped according to statistical similarity (α = 0.05). Figure S7: Post hoc pairwise comparisons of optical density (OD_600_) between AgNPs concentrations using Tukey’s honestly significant difference (HSD) test following two-way ANOVA. Figure S8: Boxplot distribution of optical density (OD_600_) as a function of AgNps concentration (25, 50, and 100 ppm). Colors indicate exposure times (0, 24, 48, and 72 h). The plot illustrates a concentration-dependent decrease in optical density across all time points, indicating enhanced antimicrobial activity at higher AgNps concentrations. Figure S9: Boxplot distribution of optical density (OD_600_) as a function of exposure time (0, 24, 48, and 72 h). Colors represent AgNps concentrations (25, 50, and 100 ppm). A pronounced reduction in optical density is observed after 24 h, followed by comparable distributions at 48 h and 72 h, suggesting a sustained antimicrobial effect over time.

## Abbreviations

The following abbreviations are used in this manuscript:

*AgNPs*	silver nanoparticles
DLS	dynamic light scattering
TEM	transmission electron microscopy
SAED	selected area electron diffraction
