# Supplementary material for: Contact-Dependent Antibacterial Performance of Silver Nanoparticles Encapsulated in Collagen-Based Gels
Source: J Funct Biomater. 2026 Mar 2;17(3):120. doi: 10.3390/jfb17030120 (PMC13027976; doi:10.3390/jfb17030120)
Supplement: Supplementary file 1 [file jfb-17-00120-s001.zip › jfb-4104044-supplementary.pdf]

Supplementary material

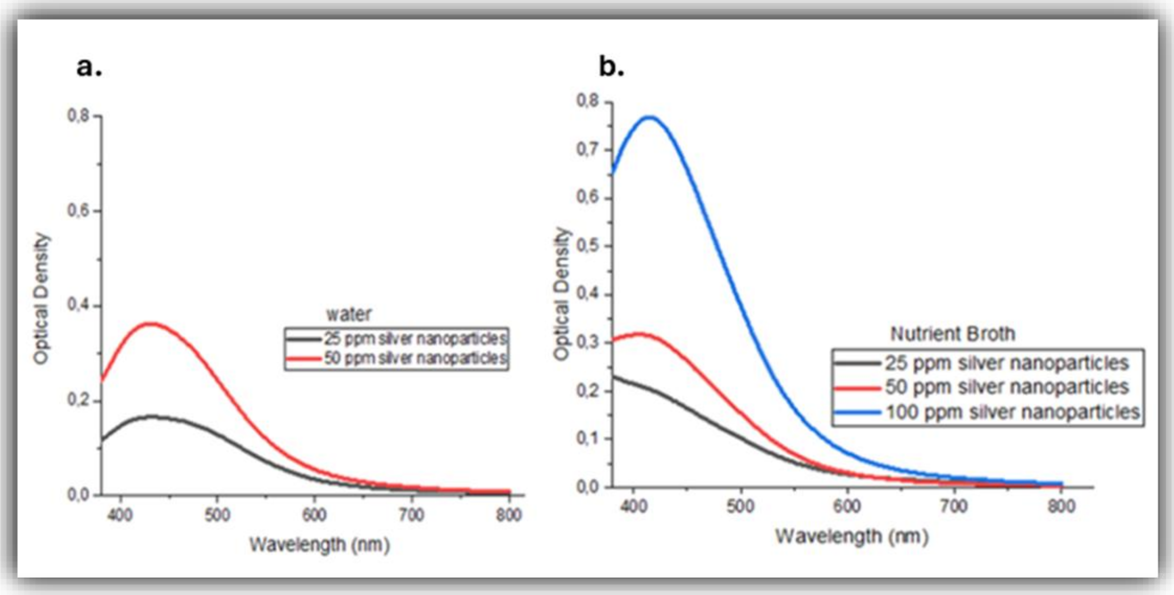

Figure S1. (a) UV-Vis of 25, 50 ppm of suspension of silver nanoparticles in water and (b) UV-Vis of 25, 50 and 100 ppm of silver nanoparticles in nutrient broth.

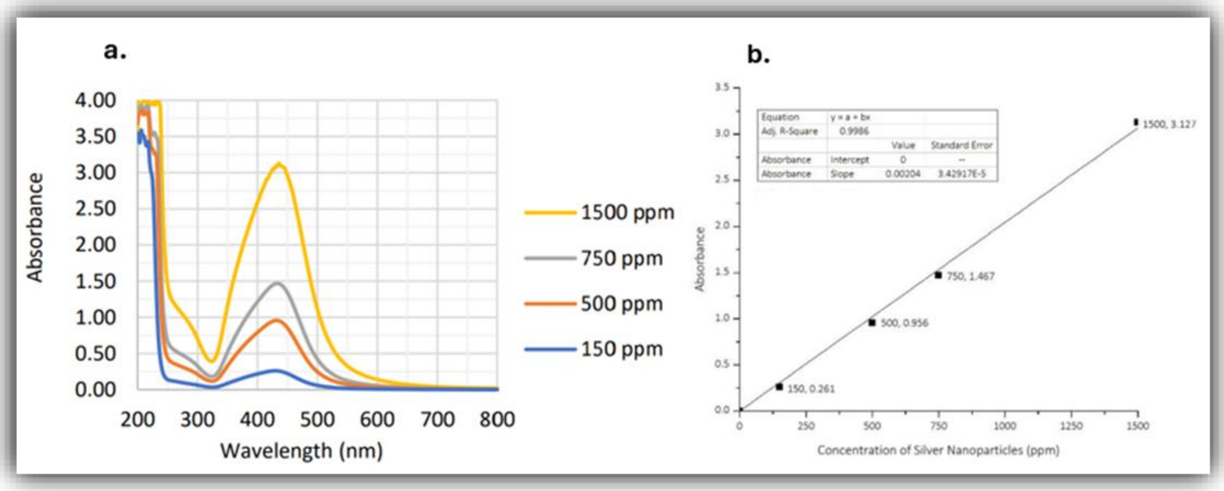

Figure S2. (a) UV-Vis of 150-1500ppm suspension of silver nanoparticles after sonication (b) Absorbance at maximum (*ca*425nm) as a function of silver nanoparticles' concentration.

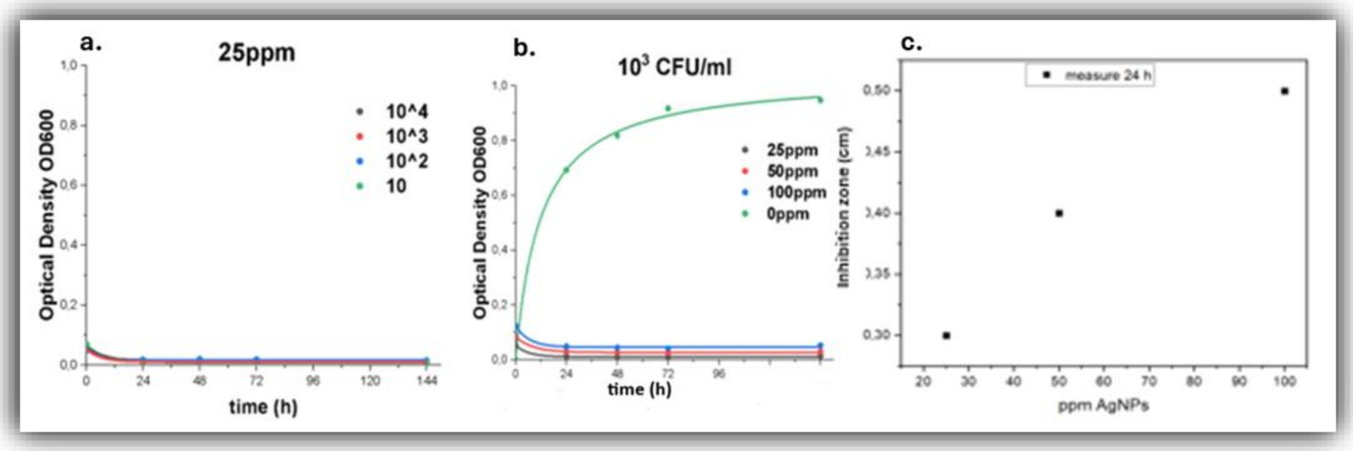

Figure S3. (a) Growth kinetics of *E. coli* in liquid substrates, followed by optical density at 600nm incubated in the presence of 25ppm AgNPs, as a function of initial bacterial load, (b)Optical density over time for diluted *E. coli* culture with initial concentration 1000 cfu/mL with AgNPs 25, 50 and 100ppm, (c) zone of inhibition in relation to concentration of AgNPs.

Tests of Between-Subjects Effects

Dependent Variable: OD_600

Source	Type III Sum of Squares	df	Mean Square	F	Sig.	Partial Eta Squared
Corrected Model	.097[a]	8	.012	20.370	<.001	.807
Intercept	.135	1	.135	227.022	<.001	.853
AgNP_ppm	.036	2	.018	29.998	<.001	.606
Groups	.006	3	.002	3.262	.031	.201
Time	.055	3	.018	31.058	<.001	.705
Error	.023	39	.001			
Total	.255	48				
Corrected Total	.120	47				

a. R Squared = .807 (Adjusted R Squared = .767)

Figure S4. Two-way analysis of variance (ANOVA) assessing the effects of AgNps concentration and exposure time on optical density (OD_{600}). Type III sums of squares, F-values, significance levels (p-values), and partial eta squared (η^2) are reported.

Multiple Comparisons

Dependent Variable: OD_600

Tukey HSD

(I) Time	(J) Time	Mean Difference (I-J)	Std. Error	Sig.	95% Confidence Interval Lower Bound	95% Confidence Interval Upper Bound
.00	24.00	.07608*	.009949	<.001	.04939	.10278
	48.00	.07850*	.009949	<.001	.05180	.10520
	72.00	.08042*	.009949	<.001	.05372	.10711
24.00	.00	-.07608*	.009949	<.001	-.10278	-.04939
	48.00	.00242	.009949	.995	-.02428	.02911
	72.00	.00433	.009949	.972	-.02236	.03103
48.00	.00	-.07850*	.009949	<.001	-.10520	-.05180
	24.00	-.00242	.009949	.995	-.02911	.02428
	72.00	.00192	.009949	.997	-.02478	.02861
72.00	.00	-.08042*	.009949	<.001	-.10711	-.05372
	24.00	-.00433	.009949	.972	-.03103	.02236
	48.00	-.00192	.009949	.997	-.02861	.02478

Based on observed means.

The error term is Mean Square(Error) = .001.

*. The mean difference is significant at the .05 level.

Figure S5. Post hoc multiple comparisons of optical density (OD_{600}) between exposure times using Tukey's honestly significant difference (HSD) test following two-way ANOVA.

OD_600

Tukey HSD[a,b]

Time	N	Subset 1	Subset 2
72.00	12	.03133	
48.00	12	.03325	
24.00	12	.03567	
.00	12		.11175
Sig.		.972	1.000

Means for groups in homogeneous subsets are displayed.
Based on observed means.
The error term is Mean Square(Error) = .001.
a. Uses Harmonic Mean Sample Size = 12.000.
b. Alpha = .05.

Figure S6. Ranking of exposure times based on homogeneous subsets of optical density (OD_{600}) identified by Tukey's HSD test. Mean values are grouped according to statistical similarity ($\alpha = 0.05$).

Multiple Comparisons

Dependent Variable: OD_600
Tukey HSD

(I) AgNP_ppm	(J) AgNP_ppm	Mean Difference (I-J)	Std. Error	Sig.	95% Confidence Interval Lower Bound	95% Confidence Interval Upper Bound
25.00	50.00	-.02106*	.008616	.049	-.04205	-.00007
	100.00	-.06537*	.008616	<.001	-.08637	-.04438
50.00	25.00	.02106*	.008616	.049	.00007	.04205
	100.00	-.04431*	.008616	<.001	-.06530	-.02332
100.00	25.00	.06537*	.008616	<.001	.04438	.08637
	50.00	.04431*	.008616	<.001	.02332	.06530

Based on observed means.
The error term is Mean Square(Error) = .001.
*. The mean difference is significant at the .05 level.

Figure S7. Post hoc pairwise comparisons of optical density (OD_{600}) between AgNPs concentrations using Tukey's honestly significant difference (HSD) test following two-way ANOVA.

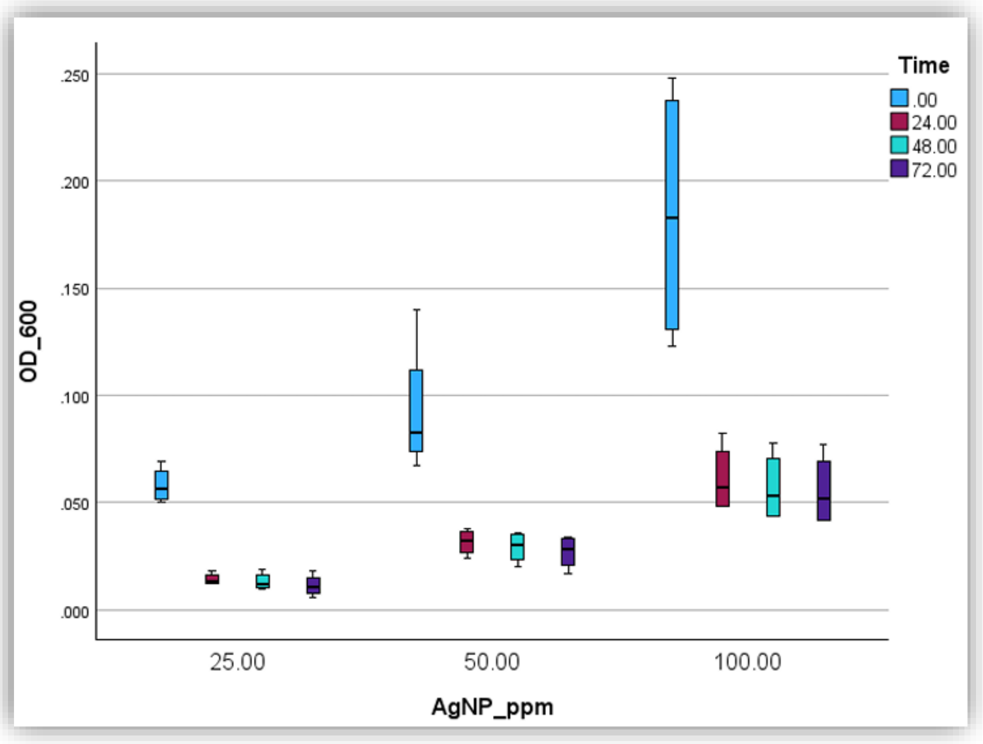

Figure S8. Boxplot distribution of optical density (OD_{600}) as a function of AgNps concentration (25, 50, and 100 ppm). Colors indicate exposure times (0, 24, 48, and 72 h). The plot illustrates a concentration-dependent decrease in optical density across all time points, indicating enhanced antimicrobial activity at higher AgNps concentrations.

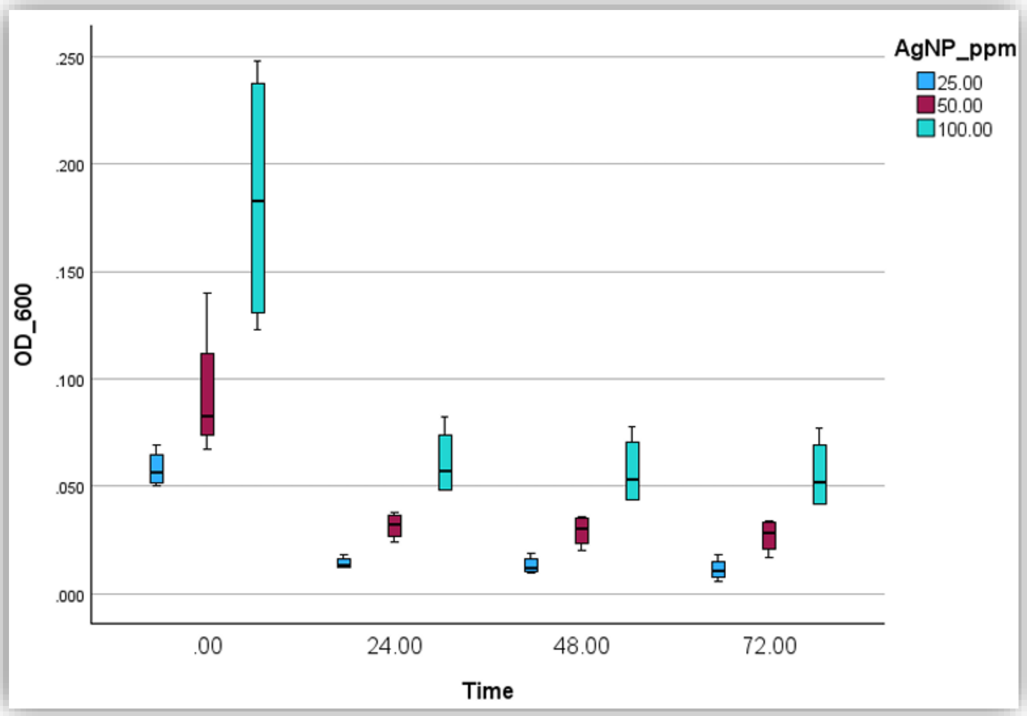

Figure S9. Boxplot distribution of optical density (OD_{600}) as a function of exposure time (0, 24, 48, and 72 h). Colors represent AgNps concentrations (25, 50, and 100 ppm). A pronounced reduction in optical density is observed after 24 h, followed by comparable distributions at 48 h and 72 h, suggesting a sustained antimicrobial effect over time.
